# Major element data, ^40^Ar/^39^Ar step-heating and step-crushing data for anorthoclase megacrysts from the Newer Volcanic Province, south-eastern Australia

**DOI:** 10.1016/j.dib.2018.06.080

**Published:** 2018-06-26

**Authors:** E.L. Matchan, D. Phillips, E. Traine, D. Zhu

**Affiliations:** School of Earth Sciences, The University of Melbourne, Parkville, VIC 3010, Australia

## Abstract

We provide the dataset associated with the research article “^40^Ar/^39^Ar ages of alkali feldspar xenocrysts constrain the timing of intraplate basaltic volcanism” Matchan et al. [1]. This dataset contains major element data for 15 large anorthoclase xenocrysts (‘megacrysts’) collected from six Pleistocene eruption centres (Mount Leura, Mount Shadwell, Mount Noorat, Mount Franklin, Lake Keilambete and The Anakies (East Cone)) in the basaltic Newer Volcanic Province of south-eastern Australia. It also contains multi-collector (Argus VI) ^40^Ar/^39^Ar step-heating for 13 of these anorthoclase megacrysts. ^40^Ar/^39^Ar vacuo step-crushing experiment data is also provided for three of these megacrysts.

**Specifications Table**

**Table:** 

Subject area	*Geology*
More specific subject area	*Geochronology*
Type of data	*Tables, figure*
How data was acquired	*Thermo Fisher Scientific Argus VI multi-collector mass spectrometer (*^*40*^*Ar/*^*39*^*Ar data); Cameca SX-50 electron microprobe (major element data)*
Data format	*Processed*
Experimental factors	*Feldspar megacryst fragments free from obvious alteration and inclusions were mounted in resin blocks, polished and carbon-coated for electron microprobe analysis*
	*Hand-picked feldspar chips were cleaned ultrasonically in demineralised water, followed by acetone, prior to neutron irradiation and*^*40*^*Ar/*^*39*^*Ar step-heating/step-crushing analyses.*
Experimental features	*Operating conditions used for electron microprobe analysis were: accelerating voltage of 15 kV, beam current of 10 nA, beam diameter of 10 μm; counting times of 20–40 s on peak positions and 10 to 40 s on two background positions located on either side of the peak position. A defocused electron beam was used to avoid loss of volatile species.*
	*For*^*40*^*Ar/*^*39*^*Ar step-heating analyses, feldspar megacryst aliquots were step-heated in vacuo using a CO*_*2*_*laser with resultant gas cleaned prior to isotopic measurement on an Argus VI multi-collector mass spectrometer.*
	*In vacuo, manual step-crushing experiments were conducted using Nupro® valves, with resultant gas cleaned prior to isotopic measurement on an Argus VI multi-collector mass spectrometer.*
Data source location	*Samples were analysed at the University of Melbourne, Parkville, Victoria, Australia.*
	*Sampling localities:*
	*The Anakies: 37°53′50′′S, 144°16′52′′E*
	*Mount Leura: 38°14′44′′S, 143°09′17′′E*
	*Mount Shadwell: 38°03′20′′S, 142°48′36′′E*
	*Mount Noorat: 8°10′42′′S, 142°56′0′′E*
	*Mount Franklin: 37°15′58′′S, 144°9′3′′E*
	*Lake Keilambete: 38°12′30′′S, 144°52′48′′E*
Data accessibility	*N/A*
Related research article	*Matchan, E.L., Phillips, D., Traine, E., Zhu, D.*^*40*^*Ar/*^*39*^*Ar ages of alkali feldspar xenocrysts constrain the timing of intraplate basaltic volcanism. Quaternary Geochronology 2018 47:14–28.*

**Value of the data**

• The ^40^Ar/^39^Ar age data for feldspar megacrysts could be compared with basalt groundmass ^40^Ar/^39^Ar age data for the same eruption centres to evaluate time elapsed between feldspar crystallization and entrainment by basaltic melt.
• Future ^40^Ar/^39^Ar dating and major element compositional studies on feldspar megacrysts (and potentially other K-bearing phases) from the Newer Volcanic province could provide insights into timeframes of megacryst formation by evaluating how many generations of megacrysts are represented at a single eruption centre.
• The ^40^Ar/^39^Ar step-heating data presented here could be used in future studies evaluating mechanisms for argon isotopic disturbance (e.g. recoil, mass fractionation) in high-temperature feldspars.

## 1 Data

Table A1 contains electron microprobe data for sixteen feldspar megacrysts. Table A2 contains processed data from ^40^Ar/^39^Ar step-heating and step-crushing experiments on feldspar megacrysts. Data have been corrected for mass spectrometer backgrounds, discrimination, radioactive decay and interference corrections. Table A3 contains processed data from ^40^Ar/^39^Ar step-heating and step-crushing experiments on feldspar megacrysts. Data have been corrected for mass spectrometer backgrounds, discrimination, and radioactive decay only. Table A4 contains processed data from ^40^Ar/^39^Ar fusion analyses of Alder Creek Rhyolite sanidine (double-grain aliquots). Data have been corrected for mass spectrometer backgrounds, discrimination, radioactive decay and interference corrections. Fig. A1 shows individual ^40^Ar/^39^Ar age spectra and inverse isochron diagrams for all analysed feldspar megacrysts.

## 2 Experimental design, materials, and methods

### 2.1 Sample preparation

Feldspar megacryst fragments free from obvious alteration and inclusions were mounted in resin blocks, polished and carbon-coated for electron microprobe analysis [AN1 (Shadwell), M51616 (Shadwell), AN2 (Noorat), M51449 (Lake Keilambete), M13963 (Franklin), M21210 (Franklin), M22049 (Anakies, East Cone), and M11753 (Anakies, East Cone)]. Doubly-polished sections (~200 μm thickness) were prepared for fluid inclusion analysis. The remaining fragments were crushed manually using a steel mortar to achieve a grain size of 1–3 mm. Approximately 100–300 mg of crushed chips with no alteration or obvious solid inclusions were hand-picked under binocular microscope. In the case of samples AN1, AN2, M21210 and M51449-b, megacryst cores were selected for processing in order to minimise argon loss effects.

Hand-picked feldspar chips were cleaned ultrasonically in demineralised water, followed by acetone. Samples were then weighed and loaded into aluminium foil packets, placed in quartz tubes (UM#50, UM#51 and UM#70) along with the flux monitor Alder Creek Rhyolite sanidine (ACRs: 1.18144 ± 0.00068 Ma [2]) and irradiated in the CLICIT facility at the Oregon State University TRIGA reactor (UM#50 - 3 MWh; UM#51 - 10 MWh; UM#70 - 0.75 MWh).

### 2.2 Mineral chemistry

Electron microprobe analysis of feldspar fragments was undertaken using a Cameca SX-50 electron microprobe at the University of Melbourne. This instrument is equipped with four vertical wavelength dispersive spectrometers and operating conditions used were: accelerating voltage of 15 kV, beam current of 10 nA, beam diameter of 10 μm; counting times of 20–40 s on peak positions and 10–40 s on two background positions located on either side of the peak position; detection limits of ≤ 400 ppm for all elements except Ba (~800 ppm). Analyses were conducted using a defocused electron beam to avoid loss of volatile species (e.g. F, Cl and K). Elemental data, relative to natural and synthetic mineral and elemental standards, are reported in Table A1.

### 2.3 ^40^Ar/^39^Ar geochronology

^40^Ar/^39^Ar analyses were undertaken in the Noble Gas Geochronology Laboratory at the University of Melbourne, using a multi-collector Thermo Fisher Scientific Argus VI mass spectrometer linked to a stainless steel gas extraction/purification line and a Photon Machines Fusions 10.6 CO_2_ laser system [2], [3]. ^36^Ar was measured using a Compact Discrete Dynode (CDD) detector, with the remaining isotopes measured on Faraday detectors with low-noise amplifiers (1 ×10^12^ Ω resistors, with the exception of the more recent UM#70 analyses, where the ^39^Ar collector was equipped with a 1 ×10^13^ Ω resistor).

Following neutron irradiation and cooling, separate aliquants of the samples were prepared for in vacuo step-crushing and detailed step-heating experiments. This approach, as opposed to two/three step-heating or direct fusion experiments, allowed for investigation of excess argon in the samples. Step-crushing experiments were undertaken on several megacrysts to directly evaluate the isotopic composition of argon trapped in defects (e.g. fluid inclusions) in the following samples: AN1 (Mount Shadwell), AN2 (Mount Noorat), M51449 (Lake Keilambete), and M21210 (Mount Franklin). An aliquant size of either 60 mg or 120 mg was loaded across a series of four modified Nupro® valves (~30 mg per valve) for in vacuo step-crushing experiments, following procedures described by Kendrick et al. (2006). Crushed samples were subsequently recovered for ^40^Ar/^39^Ar laser step-heating analysis.

Uncrushed and crushed sample aliquants (typically 70–100 mg) were loaded into custom-made copper sample holders, covered with a ZnS-glass cover slip, and placed into a stainless steel sample chamber. The sample chamber and extraction line were baked overnight at ~120 °C, and samples were outgassed at low laser-power using the 6 mm homogenised beam to remove any adsorbed atmospheric argon from grain surfaces. Air aliquots from an automated pipette system were analysed prior to sample analyses to monitor mass discrimination and detector bias. Samples were heated using the homogenized 6 mm beam. Taking into account the different low-temperature outgassing procedures, step-heating experiments (7–10 steps) were conducted over a heating interval of either 1.8–5.7 W (8–30% laser power; UM#50,51 samples), or 0.45–6.4 W (2–35% laser power; UM#70 samples). Gas introduced into the Argus VI mass spectrometer was equilibrated for 20 s, before peak centring on mass 40 (H1) and multi-collector analysis of the five argon isotopes. Peak signals were collected for a period of 300 s and regressed to the time of gas inlet. Line blanks, measured between blocks of 3–4 sample analyses, were typically 1–2 fA for ^40^Ar, compared to typical sample signal sizes of >100 fA. Line blanks were subtracted from succeeding sample results. Alder Creek Rhyolite (ACR) sanidine grains were analysed on the same system. Grains were either directly fused (35% laser power) or heated in two steps (14–18% and 35% laser power), with gas cleanup and measurement as for samples (data in Table A4). The weighted mean ^40^Ar/^39^Ar result was used to calculate the *J*-value, assuming an ACR sanidine age of 1.18144 ± 0.00068 Ma (95% CI) [2].

Due to the short irradiation times it was not feasible to include Ca/K/Cl salts/glasses in the same packages as the samples. Therefore, correction factors relate to K-glass and Ca-salts contained in packages irradiated close in time in the CLICIT facility. Interference correction values used for UM#50 and UM#51 were: (^36^Ar/^37^Ar)_Ca_ = (2.700 ± 0.020) × 10^−^^4^; (^39^Ar/^37^Ar)_Ca_ = (7.600 ± 0.090) × 10^−^^4^; (^40^Ar/^39^Ar)_K_ = (7.30 ± 0.97) × 10^−4^; (^38^Ar/^39^Ar)_K_ = (1.300 ± 0.050) × 10^-2^. Interference correction values used for UM#70 were: (^36^Ar/^37^Ar)_Ca_ = (2.5782 ± 0.0018) × 10^−4^; (^39^Ar/^37^Ar)_Ca_ = (6.562 ± 0.016) × 10^−4^; (^40^Ar/^39^Ar)_K_ = (1.00 ± 0.05) × 10^−10^; (^38^Ar/^39^Ar)_K_ = (1.2246 ± 0.0028) × 10^−2^. Note that the (^40^Ar/^39^Ar)_K_ ratio used for UM#70 (1.00 ± 0.05) × 10^−^^10^ was an estimate (due to difficulties measuring the [^40^Ar/^39^Ar]_K_ ratio at the time); however, employing the value used for earlier irradiation batches (7.30 × 10^−^^4^) has an insignificant effect on the results. The decay constants of Steiger and Jäger [4] were assumed.

Apparent ages (Table A2) were calculated based on: (i) a default atmospheric composition (298.56 [5]); and (ii) the corresponding inverse isochron determined (^40^Ar/^36^Ar)_i_ value (Fig. A1). In the latter case, uncertainties in (^40^Ar/^36^Ar)_i_ ratios were included in reported age uncertainties. Age spectra and inverse isochron diagrams (Fig. A1) were generated using ISOPLOT/Ex.3.75 [6]. Plateau ages are defined as including at least 50% of the ^39^Ar, distributed over a minimum of three contiguous steps and with ^40^Ar*/^39^Ar ratios within agreement of the mean at the 95% confidence level (e.g. [7]). Plateau ages were calculated only in cases where (^40^Ar/^36^Ar)_i_ values were within error of the atmospheric value and, by convention, do not include the uncertainty in the air ratio (298.56 ± 0.62 (0.21%), 2*σ* [5]).
