# Supplementary material for: Major element data, 40Ar/39Ar step-heating and step-crushing data for anorthoclase megacrysts from the Newer Volcanic Province, south-eastern Australia
Source: Data Brief. 2018 Jun 26;19:1847–51. doi: 10.1016/j.dib.2018.06.080 (PMC6141373; doi:10.1016/j.dib.2018.06.080)
Supplement: Supplementary file 1 — Supplementary material [file mmc1.docx]

02/05/18

**Declaration of Interest**

*Article submitted to Data In Brief: Major element data, ^40^Ar/^39^Ar step-heating and step-crushing data for anorthoclase megacrysts from the Newer Volcanic Province, south-eastern Australia*

I declare that there are no conflicts of interest for this work.

Yours sincerely,

Erin Matchan (corresponding author), David Phillips, Esther Traine, Dongliang Zhu.
