# Supplementary material for: Major element data, 40Ar/39Ar step-heating and step-crushing data for anorthoclase megacrysts from the Newer Volcanic Province, south-eastern Australia
Source: Data Brief. 2018 Jun 26;19:1847–51. doi: 10.1016/j.dib.2018.06.080 (PMC6141373; doi:10.1016/j.dib.2018.06.080)
Supplement: Supplementary file 2 — Supplementary material [file mmc2.pdf]

Fig A1 Individual age spectra and inverse isochron diagrams for anorthoclase megacrysts as generated using Isoplot. Uncertainties for Model 1 fits are 2σ and for Model 2 are 95% CI.

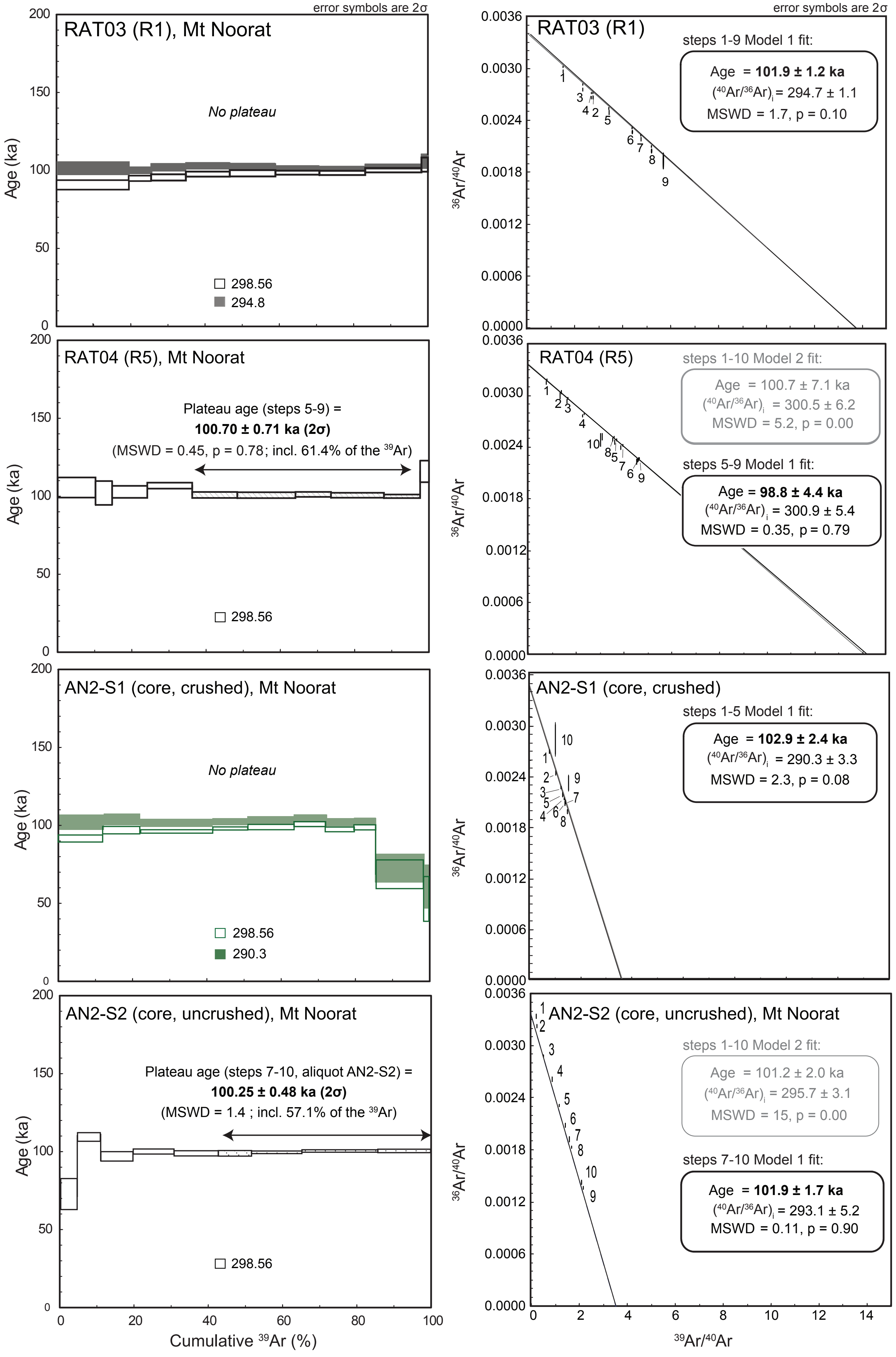

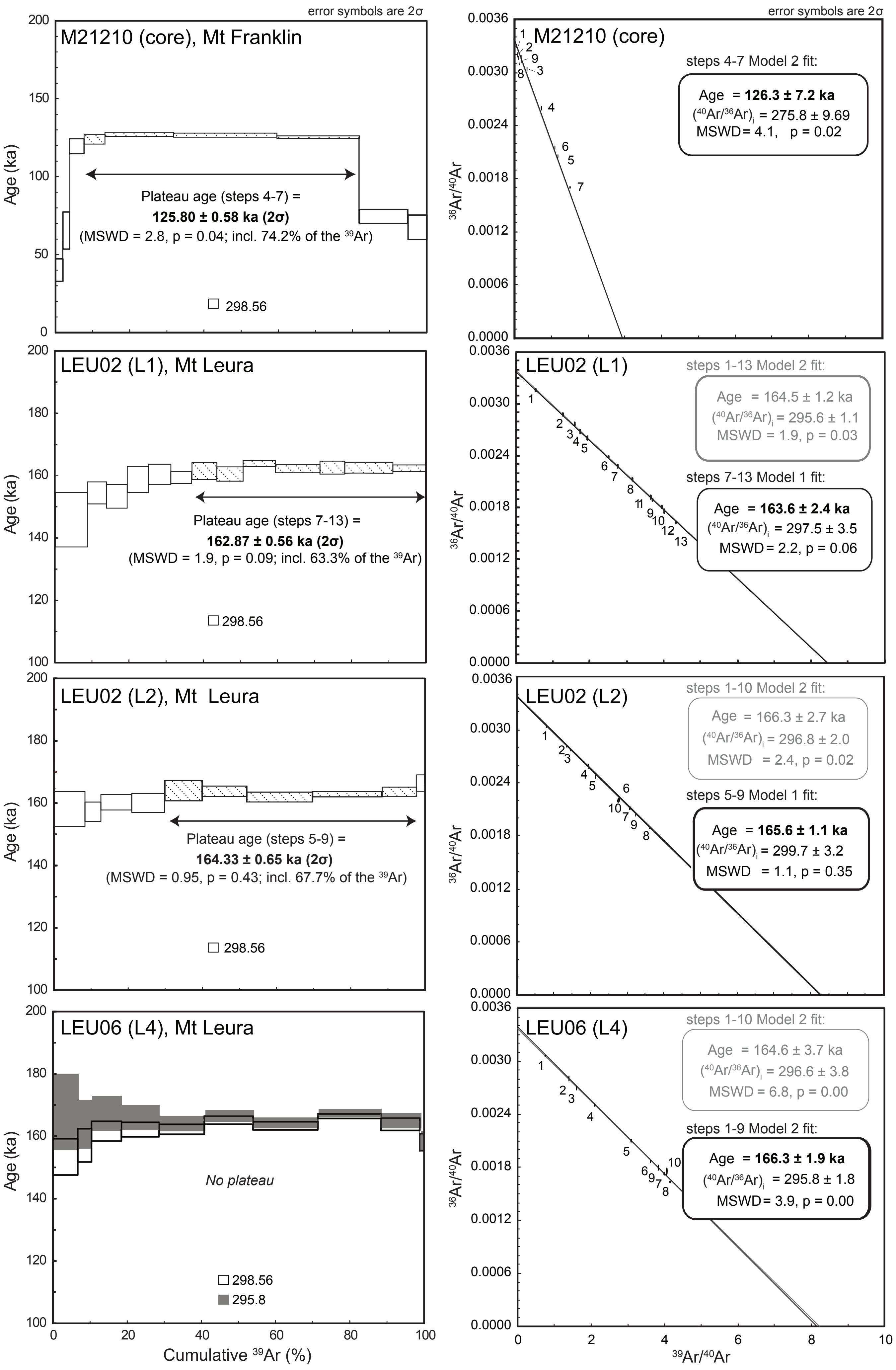
error symbols are 2σ
M21210 (core), Mt Franklin
Plateau age (steps 4-7) =
125.80 ± 0.58 ka (2σ)
(MSWD = 2.8, p = 0.04; incl. 74.2% of the 39Ar)
298.56
M21210 (core)
steps 4-7 Model 2 fit:
Age = 126.3 ± 7.2 ka
(40Ar/36Ar)i = 275.8 ± 9.69
MSWD = 4.1, p = 0.02
LEU02 (L1), Mt Leura
Plateau age (steps 7-13) =
162.87 ± 0.56 ka (2σ)
(MSWD = 1.9, p = 0.09; incl. 63.3% of the 39Ar)
298.56
LEU02 (L1)
steps 1-13 Model 2 fit:
Age = 164.5 ± 1.2 ka
(40Ar/36Ar)i = 295.6 ± 1.1
MSWD = 1.9, p = 0.03
steps 7-13 Model 1 fit:
Age = 163.6 ± 2.4 ka
(40Ar/36Ar)i = 297.5 ± 3.5
MSWD = 2.2, p = 0.06
LEU02 (L2), Mt Leura
Plateau age (steps 5-9) =
164.33 ± 0.65 ka (2σ)
(MSWD = 0.95, p = 0.43; incl. 67.7% of the 39Ar)
298.56
LEU02 (L2)
steps 1-10 Model 2 fit:
Age = 166.3 ± 2.7 ka
(40Ar/36Ar)i = 296.8 ± 2.0
MSWD = 2.4, p = 0.02
steps 5-9 Model 1 fit:
Age = 165.6 ± 1.1 ka
(40Ar/36Ar)i = 299.7 ± 3.2
MSWD = 1.1, p = 0.35
LEU06 (L4), Mt Leura
No plateau
298.56
295.8
LEU06 (L4)
steps 1-10 Model 2 fit:
Age = 164.6 ± 3.7 ka
(40Ar/36Ar)i = 296.6 ± 3.8
MSWD = 6.8, p = 0.00
steps 1-9 Model 2 fit:
Age = 166.3 ± 1.9 ka
(40Ar/36Ar)i = 295.8 ± 1.8
MSWD = 3.9, p = 0.00
Age (ka)
Cumulative 39Ar (%)
36Ar/40Ar
39Ar/40Ar

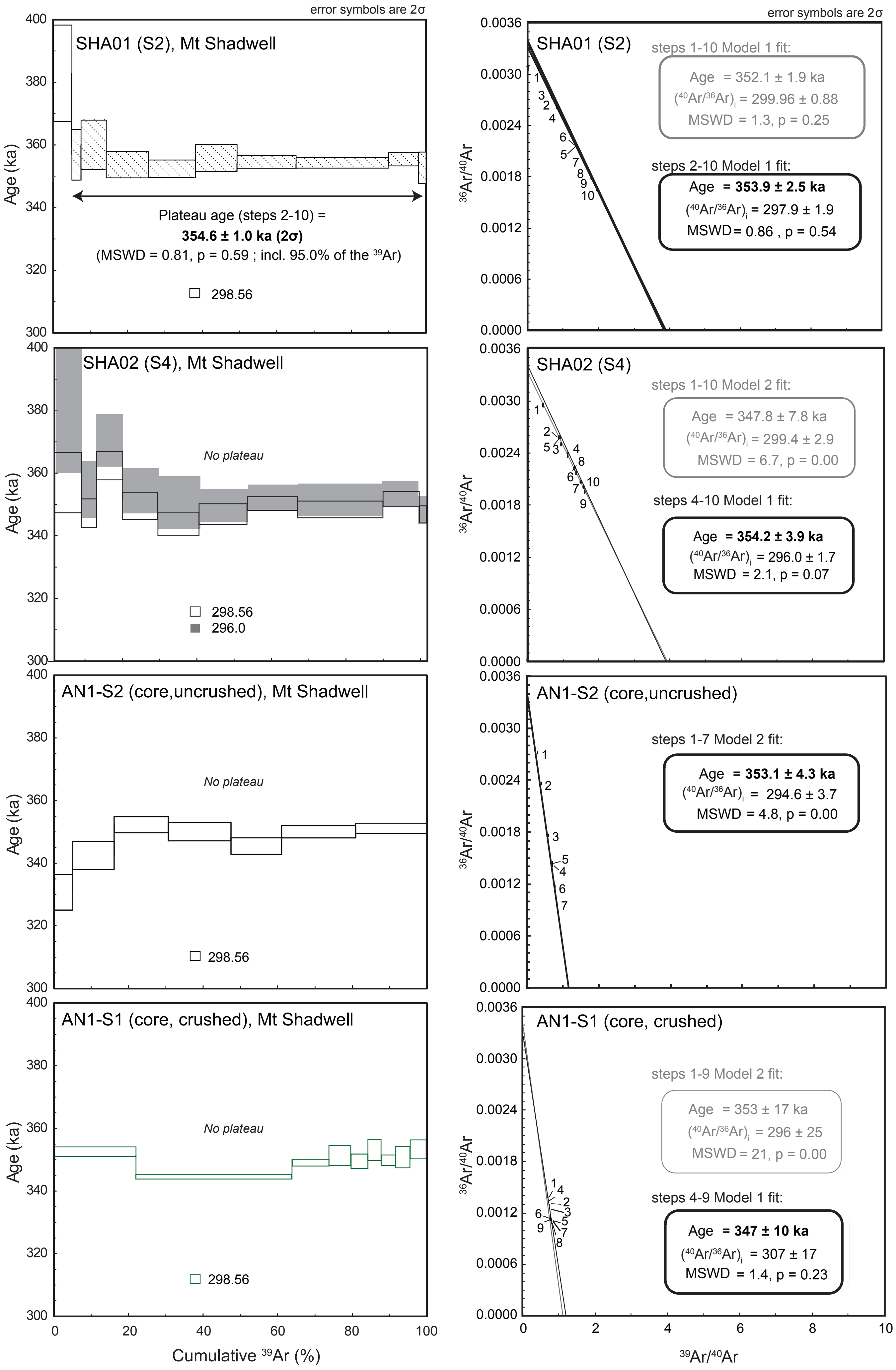
error symbols are 2σ
SHA01 (S2), Mt Shadwell
Age (ka)
Plateau age (steps 2-10) =
354.6 ± 1.0 ka (2σ)
(MSWD = 0.81, p = 0.59 ; incl. 95.0% of the 39Ar)
298.56
error symbols are 2σ
SHA01 (S2)
steps 1-10 Model 1 fit:
Age = 352.1 ± 1.9 ka
(40Ar/36Ar)i = 299.96 ± 0.88
MSWD = 1.3, p = 0.25
steps 2-10 Model 1 fit:
Age = 353.9 ± 2.5 ka
(40Ar/36Ar)i = 297.9 ± 1.9
MSWD= 0.86 , p = 0.54
36Ar/40Ar
SHA02 (S4), Mt Shadwell
No plateau
298.56
296.0
SHA02 (S4)
steps 1-10 Model 2 fit:
Age = 347.8 ± 7.8 ka
(40Ar/36Ar)i = 299.4 ± 2.9
MSWD = 6.7, p = 0.00
steps 4-10 Model 1 fit:
Age = 354.2 ± 3.9 ka
(40Ar/36Ar)i = 296.0 ± 1.7
MSWD = 2.1, p = 0.07
AN1-S2 (core,uncrushed), Mt Shadwell
No plateau
298.56
AN1-S2 (core,uncrushed)
steps 1-7 Model 2 fit:
Age = 353.1 ± 4.3 ka
(40Ar/36Ar)i = 294.6 ± 3.7
MSWD = 4.8, p = 0.00
AN1-S1 (core, crushed), Mt Shadwell
No plateau
298.56
AN1-S1 (core, crushed)
steps 1-9 Model 2 fit:
Age = 353 ± 17 ka
(40Ar/36Ar)i = 296 ± 25
MSWD = 21, p = 0.00
steps 4-9 Model 1 fit:
Age = 347 ± 10 ka
(40Ar/36Ar)i = 307 ± 17
MSWD = 1.4, p = 0.23
Cumulative 39Ar (%)
39Ar/40Ar

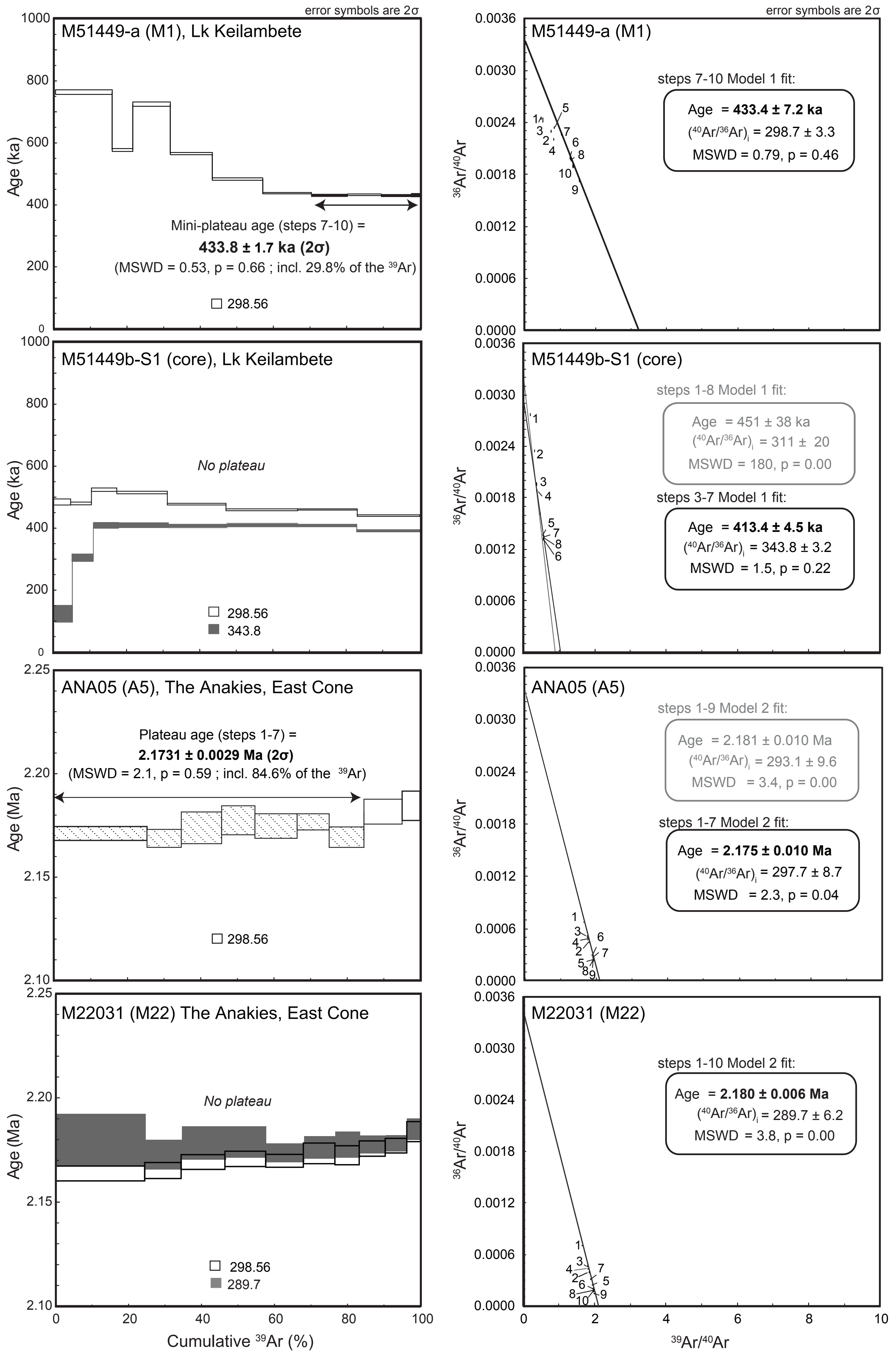
error symbols are 2σ
M51449-a (M1), Lk Keilambete
Age (ka)
Mini-plateau age (steps 7-10) =
433.8 ± 1.7 ka (2σ)
(MSWD = 0.53, p = 0.66 ; incl. 29.8% of the 39Ar)
298.56
error symbols are 2σ
M51449-a (M1)
36Ar/40Ar
steps 7-10 Model 1 fit:
Age = 433.4 ± 7.2 ka
(40Ar/36Ar)i = 298.7 ± 3.3
MSWD = 0.79, p = 0.46
M51449b-S1 (core), Lk Keilambete
No plateau
298.56
343.8
M51449b-S1 (core)
steps 1-8 Model 1 fit:
Age = 451 ± 38 ka
(40Ar/36Ar)i = 311 ± 20
MSWD = 180, p = 0.00
steps 3-7 Model 1 fit:
Age = 413.4 ± 4.5 ka
(40Ar/36Ar)i = 343.8 ± 3.2
MSWD = 1.5, p = 0.22
ANA05 (A5), The Anakies, East Cone
Age (Ma)
Plateau age (steps 1-7) =
2.1731 ± 0.0029 Ma (2σ)
(MSWD = 2.1, p = 0.59 ; incl. 84.6% of the 39Ar)
298.56
ANA05 (A5)
steps 1-9 Model 2 fit:
Age = 2.181 ± 0.010 Ma
(40Ar/36Ar)i = 293.1 ± 9.6
MSWD = 3.4, p = 0.00
steps 1-7 Model 2 fit:
Age = 2.175 ± 0.010 Ma
(40Ar/36Ar)i = 297.7 ± 8.7
MSWD = 2.3, p = 0.04
M22031 (M22) The Anakies, East Cone
No plateau
298.56
289.7
Cumulative 39Ar (%)
M22031 (M22)
steps 1-10 Model 2 fit:
Age = 2.180 ± 0.006 Ma
(40Ar/36Ar)i = 289.7 ± 6.2
MSWD = 3.8, p = 0.00
39Ar/40Ar
